# Topological Bias in Distance-Based Phylogenetic Methods: Problems with Over- and Underestimated Genetic Distances

**Published:** 2007-02-26

**Authors:** Xuhua Xia

**Affiliations:** Department of Biology, University of Ottawa, Ottawa, Ontario, Canada

**Keywords:** topological bias, minimum evolution, least-squares method, Fitch-Margoliash

## Abstract

I show several types of topological biases in distance-based methods that use the least-squares method to evaluate branch lengths and the minimum evolution (ME) or the Fitch-Margoliash (FM) criterion to choose the best tree. For a 6-species tree, there are two tree shapes, one with three cherries (a cherry is a pair of adjacent leaves descending from the most recent common ancestor), and the other with two. When genetic distances are underestimated, the 3-cherry tree shape is favored with either the ME or FM criterion. When the genetic distances are overestimated, the ME criterion favors the 2-cherry tree, but the direction of bias with the FM criterion depends on whether negative branches are allowed, i.e. allowing negative branches favors the 3-cherry tree shape but disallowing negative branches favors the 2-cherry tree shape. The extent of the bias is explored by computer simulation of sequence evolution.

## Introduction

Topological bias of phylogenetic methods has been noted a long time ago as a potential source of bias in the study of speciation processes (Huelsenbeck and Kirkpatrick, 1996). Understanding the bias in different phylogenetic methods can help us explain previously unexpected observations in phylogenetic studies (Bruno and Halpern, 1999; Hillis, 1998; Huelsenbeck, 1998; Purvis and Agapow, 2002; Swofford et al. 2001). For example, a rooted tree with eight operational taxonomic units (OTUs) has many fewer possible perfectly symmetrical trees than maximally asymmetric trees. Consequently, a phylogenetic algorithm that picks up random trees, or even a good phylogenetic algorithm working on data that have already lost almost all the phylogenetic information such as extremely diverged sequences, would be more likely to end up with a maximally asymmetrical tree than a perfectly symmetrical tree (Huelsenbeck and Kirkpatrick, 1996).

Topological bias may confound the evaluation of the relative performance of phylogenetic methods in studies (e.g. Yang, 1997) that use a model tree to simulate sequence evolution and evaluate phylogenetic algorithms by checking which one is the most efficient in recovering the model tree. For example, if a 4-OTU model tree has two sister OTUs with long branches, then phylogenetic algorithms, especially the maximum parsimony method, that suffers from the long-branch attraction problem will tend to be the most efficient in recovering the known tree and may be misconstrued to be the best algorithm (Bruno and Halpern, 1999).

Here I report several kinds of topological biases in phylogenetic reconstruction by the distance-based methods based on the minimum evolution or Fitch-Margoliash criterion in selecting the best topology. The distance-based methods for phylogenetic reconstruction have several advantages over maximum parsimony and maximum likelihood methods. First, they are typically fast. Second, one can implement complicated substitution models such as those underlying the paralinear and Log-Det distances that would be difficult to implement in a maximum likelihood framework, although such attempt has been made recently (Jayaswal et al. 2005). Third, they appear to suffer less from the inconsistencies reported for maximum parsimony methods. Forth, they are better than maximum parsimony methods in estimating divergence time because of its model-based correction for multiple hits. For these and perhaps many other reasons, the distance-based methods have been used widely in molecular phylogenetics, especially with a large number of OTUs or in large-scale simulations (e.g. Xia et al. 2003b). In particular, the simplicity of the distance-based methods can often allow researchers to identify potential bias in reconstructed phylogenetic trees more readily than other methods (e.g. Xia et al. 2003a).

Topological bias associated with the least-squares method and the minimum evolution (ME) criterion has previously been illustrated with four OTUs (operational taxonomic units) (Xia, 2006). Here I further explore the bias with six OTUs and with both the ME and the Fitch-Margoliash (FM) criterion. The reason for using more than four or five species is because a 6-OTU tree has two tree shapes (Felsenstein, 2004, p. 33) whereas a 4-OTU or 5-OTU tree has only one tree shape and consequently is not useful to explore bias associated with tree shapes.

I will first briefly describe the statistical features common to frequently used distance-based methods and illustrate the systematic bias shared among these distance-based methods when the minimum evolution (ME) or Fitch-Margoliash (FM) criterion is used in choosing the best tree. This is followed by computer simulations to explore the extent of the bias.

## Mechanistic Illustration of the Bias

**Figure 1:**
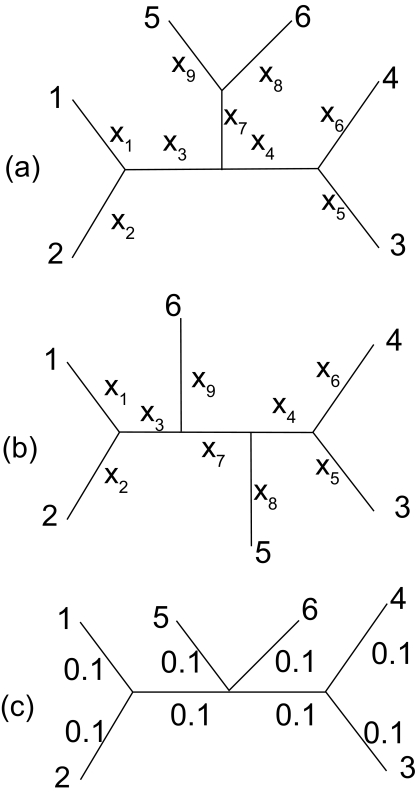
Two contrasting topologies (a) and (b) with six OTUs, together with a third topology (c) which is the intermediate of the two. Topologies A and B converges to Topology C when *x*_7_ approaches zero.

Let us start with the two contrasting topologies A and B (Fig. 1). Designate *D_ij_* as the genetic distance between OTUs *i* and *j*. The least-squares estimate of *x_i_* for Topology A (Fig. 1a) is:

(1) $$\begin{array}{l} x_{1}=\frac{D_{13}+D_{14}+D_{15}+D_{16}}{8}+\frac{D_{12}}{2}\\ -\frac{D_{23}+D_{24}+D_{25}+D_{26}}{8}\\ \cdots \\ x_{9}=\frac{D_{15}+D_{25}+D_{35}+D_{45}}{8}+\frac{D_{56}}{2}\\ -\frac{D_{16}+D_{26}+D_{36}+D_{46}}{8} \end{array}$$

The *x_i_* values for Topology B (Fig. 1b) are,

(2) $$\begin{array}{l} x_{1}=\frac{D_{13}+D_{14}+D_{15}+D_{16}}{8}+\frac{D_{12}}{2}\\ -\frac{D_{23}+D_{24}+D_{25}+D_{26}}{8}\\ \cdots \\ x_{9}=\frac{D_{36}+D_{46}+D_{56}}{6}+\frac{D_{16}+D_{26}}{4}\\ -\frac{D_{13}+D_{14}+D_{15}+D_{23}+D_{24}+D_{25}}{12} \end{array}$$

To save space in writing equations, I will equate:

(3) $$\begin{array}{l} A=D_{15}+D_{25}+D_{36}+D_{46}\\ B=D_{13}+D_{14}+D_{23}+D_{24}\\ C=D_{16}+D_{26}+D_{35}+D_{45} \end{array}$$

According to the minimum evolution (ME) criterion, the best tree is the one with the shortest tree length (TL). Designating the tree length of Topology A and Topology B as *TL_a_* and *TL_b_*, respectively, we have:

(4) $$\begin{array}{l} {TL}_{a}=\sum_{i=1}^{9}x_{i}=\frac{B+A+D_{25}+D_{26}+D_{35}+D_{45}}{8}\\ +\frac{D_{12}+D_{34}+D_{56}}{2}\\ {TL}_{b}=\frac{B}{18}+\frac{5A}{36}+\frac{C}{4}+\frac{D_{12}+D_{34}}{2}+\frac{2D_{56}}{9} \end{array}$$

**Figure 2:**
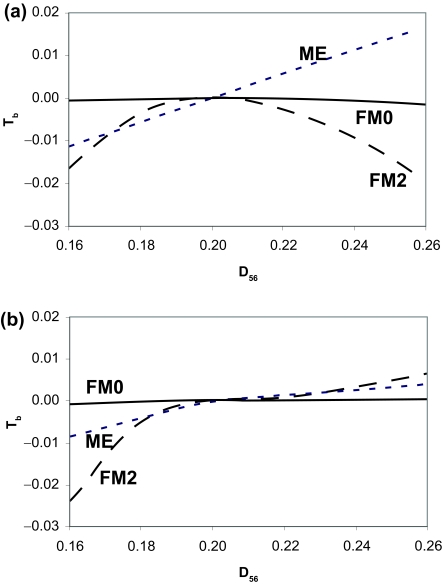
Comparison between allowing (a) and disallowing (b) negative branch lengths, given that the true tree is Topology C in Fig. 1 with all branch lengths set to 0.1 and that *D*_56_ is the only genetic distance estimated inaccurately. *T_b.ME_*, *T_b.FM_*_0_ and *T_bFM_*_2_ are explained in the text.

The tendency of favoring Topology B, measured as (*TL_a_* – *TL_b_*), is

(5) $$T_{b.ME}={TL}_{a}-{TL}_{b}=\frac{5D_{56}}{18}+\frac{5B}{72}-\frac{A}{72}-\frac{C}{8}$$

which measures the tendency to choose Topology B over Topology A. In other words, we choose Topology B if *T_b.ME_* > 0 or Topology A if *T_b.ME_* < 0. When *T_b.ME_* = 0, the two trees are equally good based on the ME criterion. *T_b.ME_* is expected to be 0 in two situations: (1) when *x*_7_ = 0 in Topology A and Topology B in Fig. 1 in which case the two topologies converge to Topology C in Fig. 1c, and (2) when sequences experienced full substitution saturation so that all *D_ij_* values are expected to be the same.

For the BME algorithm (Desper and Gascuel, 2002), the corresponding equation (derived by O. Gascuel, pers. comm.) is

(6) $$T_{b.BME}=\frac{4D_{56}+B-2C}{8}$$

It is interesting to note that, although equation (6) differ in form from equation (5), *T_b.BME_* is expected to be 0 in two identical situations mentioned in the previous paragraph: (1) when *x*_7_ = 0 in Topology A and Topology B in Fig. 1 and (2) when sequences experienced full substitution saturation so that all *D_ij_* values are expected to be the same.

The FM criterion for choosing the best tree is based on

(7) $$SS=\sum_{i}^{N_{OTU}}\sum_{j,j\neq i}^{N_{OTU}}\frac{{\left(D_{ij}-D_{ij}^{\prime }\right)}^{2}}{D_{ij}^{P}}$$

where *N_OTU_* is the number of OTUs. It is important not to confuse the FM method for evaluating branch lengths with the FM criterion for choosing the best tree. The branch length of a particular topology can be evaluated by either the least-squares method or the FM method, and the best tree can be chosen based on either the ME criterion which takes the shortest tree as the best tree or the FM criterion which takes the tree with smallest SS as the best tree.

In the FITCH program in PHYLIP, the default value for P in equation (7) is 2 but it can be 0 or any other value. Here I consider only two cases, with P = 0 (which means that the denominator will always be 1) and P = 2 (the default in PHYLIP’s FITCH program).

When P = 0, the expressions for SS in equation (7) are relatively simple for Topology A and Topology B, and their difference can be written as

(8) $$\begin{array}{l} T_{b.FM0}={SS}_{a}-{SS}_{b}={\left(A-3C\right)}^{2}-2{\left(B+4D_{56}\right)}^{2}\\ +4A(2B+8D_{56}-3C) \end{array}$$

where the subscript FM0 in *T_b.FM_*_0_ indicates the FM criterion with P = 0 and A, B, and C are defined in equation (3). The interpretation of *T_b.FM_*_0_ is the same as *T_b.ME_* in equation (7). That is, we choose Topology B if *T_b.FM_*_0_ > 0 or Topology A if *T_b.FM_*_0_ < 0. When *T_b.FM_*_0_ = 0, the two trees are equally good based on the FM0 criterion. I will use *T_b.FM_*_2_ to represent (*SS_s_*–*SS_a_*) for P = 2 (which is the default in PHYLIP’s FITCH program). The algebraic expression for *T_b.FM_*_2_ is cumbersome to write down and only the computational results will be presented and compared with *T_b.ME_* and *T_b.FM_*_0_.

Consider first Topology C in Fig. 1c with all branch lengths equal to 0.1. Note that Topology A and Topology B converges to Tree C when *x*_7_ approaches 0. When there is no error in estimating *D_ij_*, then *T_b.ME_*, *T_b.FM_*_0_ and *T_b.FM_*_2_ (not shown) are all equal to 0 for Topology C, which is expected because Topology C is exactly intermediate between Topology A and Topology B. It is known that, if *D_ij_* values are estimated accurately, then the application of the ME criterion does not have bias favoring any particular topology (Bryant and Wadell, 1998; Rzhetsky and Nei, 1993).

I now consider a special case when *D*_56_ is underestimated or overestimated, i.e. when the estimated *D*_56_ is different from the true value of 0.2 (Fig. 1c). In reality, because of the shared branches between some distances, the error will not be limited to just one distance. However, examining this special case will shed light on various aspects of the topological biases arising from the application of the ME and the FM criteria.

### Negative branch lengths allowed

#### ME criterion

*T_b.ME_* in equation (5) increases linearly with the overestimation of *D*_56_ (Fig. 2). When *D*_56_ is underestimated, *T_b.ME_* is smaller than 0 and Topology A will be the ME tree although the true tree is Topology C. When *D*_56_ is overestimated, *T_b.ME_* will be greater than 0 and Topology B will be the ME tree. The p distance, as well as other distances based on simple substitution models such as the JC69 model, tend to underestimate the true distance and will consequently tend to favor Topology A against Topology B. This suggests that some proposed guidelines favoring the use of simple distances (Nei, 1996; Nei and Kumar, 2000, pp. 112–113) may not be appropriate because they tend to favor Topology A against Topology B. In contrast, gamma-corrected distances, especially those assuming a large proportion of invariant sites, will tend to overestimate the true distance and will consequently favor Topology B against Topology A. This may be partially responsible for the poor performance of the HKY gamma distance with the ME criterion (Takahashi and Nei, 2000) because their simulated sequences are short, leading to large variances associated with *D_ij_* and frequent underestimation or overestimation of *D_ij_*.

#### FM criterion

Topology A is always favored (Fig. 2a). This is easy to understand. When *D*_56_ is underestimated, the least-squares method will find a positive *x*_7_ so that *SS_a_* in equation (7) is 0 (Table 1) and is always smaller than *SS_b_*. So *T_b.FM_*_0_ is negative and Topology A is the best. When *D*_56_ is overestimated, the least-squares method will find a negative *x*_7_ so that *SS_a_* is again 0 (Table 1) and smaller than *SS_b_*. So again *T_b.FM_*_0_ is negative and Topology A is the best. This suggests that the topological bias associated with the overestimation of *D*_56_ may be alleviated by disallowing negative branches. Disallowing negative branches is the default in PHYLIP’s FITCH program and MEGA, as well as many others. Previous simulations have shown such treatment to significantly improve the performance of distance-based methods (Kuhner and Felsenstein, 1994).

### Negative branch lengths not allowed

With Topology A, when *D*_56_ is underestimated, there is no negative branch length when branch lengths are evaluated by the least-squares method, so disallowing negative branch lengths has no effect on *SS_a_*. However, with Topology B, *x*_7_ may become negative with an underestimated *D*_56_, so disallowing negative branch lengths will affect *SS_b_*. Similarly, when *D*_56_ is overestimated, disallowing negative branch lengths will not affect *SS_b_* because all branch lengths for Topology B will be positive from the least-squares method. However, the overestimation of *D*_56_ will leads to a negative *x*_7_ for Topology A. So *SS_a_* will be affected when negative branch lengths are not allowed. I numerically illustrate below the effect of disallowing negative branch lengths.

The common treatment of negative branches is to set them to zero and re-estimated the length of other branches. Thus, when *D*_56_ is underestimated leading to a negative *x*_7_ in Topology B, we will set *x*_7_ = 0 and re-estimate the lengths of other branches in Topology B. Similarly, when *D*_56_ is overestimated leading to a negative *x*_7_ in Topology A, we will again set *x*_7_ = 0 and re-estimate the lengths of other branches in Topology A. Note that here we do not need the iterative method for estimating branch lengths with the weighted least-squares method (Felsenstein, 1997). The re-estimation results in two sets of formulae of *T_b.ME_*, *T_b.FM_*_0,_ and *T_b.FM_*_2_, with one set for underestimated *D*_56,_ and another for overestimated *D*_56_.

#### Underestimation of *D*_56_

The mathematical expressions of *T_b.ME_* and *T_b.FM_*_0_ are written below (but that for *T_b.FM_*_2_ is cumbersome and only its numerical result will be presented for comparison):

(9) $$\begin{array}{l} T_{b.ME,D_{56\;\leq \;0.2}}=\frac{3D_{56}}{14}+\frac{3(B-A-C)}{56}\\ T_{b.FM0,D_{56\;\leq \;0.2}}=-\frac{4D_{56}^{2}}{7}-\frac{2D_{56}(B-A-C)}{7}\\ -\frac{{\left(B-A-C\right)}^{2}}{28} \end{array}$$

There are three points worth highlighting. First, both *T_b.ME, D_*_56≤0.2_ and *T_b.FM_*_0,_ *_D_* _56≤0.2_ approach zero when *D*_56_ approaches the true value of 0.2, which is expected because the ME and the FM criteria are not biased when *D_ij_* values are accurately estimated (Bryant and Wadell, 1998; Rzhetsky and Nei, 1993). Second, *T_b.ME, D_*_56≤0.2_ is a linearly increasing function of the underestimated *D*_56_ with a slope of 3/14 in equation (9), which is smaller than the slope of 5/18 in equation (5). This means that the bias in favor of Topology A with an underestimated *D*_56_ is less serious when negative branch lengths are not allowed than when negative branch lengths are allowed. Third, *T_b.FM_*_0_ has a negative quadratic term such that *T_b.FM_*_0_ will approach zero asymptotically, instead of linearly as *T_b.ME_*, when *D*_56_ approaches the true value of 0.2. In particular, when *D*_56_ is underestimated, disallowing negative branch lengths does not help with the FM criterion as we can see from Fig. 2. The topological bias for *D*_56_ < 0.2 is in fact greater when negative branches are not allowed (Fig. 2b) than when negative branches are allowed (Fig. 2a).

#### Overestimation of *D*_56_

The results are quite different when *D*_56_ is overestimated. Now we have

(10) $$\begin{array}{l} T_{b.ME,D_{56}\geq 0.2}=-\frac{C}{14}+\frac{5A}{126}+\frac{B}{63}+\frac{4D_{56}}{63}\\ T_{b.FM0,D_{56}\geq 0.2}=-\frac{8D_{56}^{2}}{63}+\left(\frac{10A}{63}-\frac{2C}{7}+\frac{4B}{63}\right)D_{56}\\ +\frac{{\left(9C-5A-2B\right)}^{2}}{504} \end{array}$$

There are again three points worth highlighting. First, *T_b.ME_* increases linearly (i.e. Topology B will be increasingly favored) with overestimation of *D*_56_. However, the slope ( = 4/63) is much smaller than that for underestimated *D*_56_ shown in equation (9) where the slope is 3/14. This means that the topological bias associated with overestimating *D*_56_ will not be as serious as underestimating *D*_56_ when negative branch lengths are not allowed. This suggests that, when negative branch lengths are not allowed, overestimation of *D_ij_* is not as problematic as underestimation of *D_ij_*. Therefore, the advice of using overly simple distances that tend to underestimation *D_ij_* (Nei, 1996; Nei and Kumar, 2000, pp. 112–113) should be taken with caution. Second, for *T_b.FM_*_0_, contrary to the negative quadratic term of *D*_56_ in equation (8) where negative branch lengths are allowed, the quadratic term of *D*_56_ in equation (10) is positive. This means that the application of the FM criterion will no longer favor Topology A against Topology B as it did before when negative branch lengths are allowed. Instead, the topological bias has changed direction when we change from allowing negative branch lengths to disallowing negative branch lengths.

The topological bias associated with the inaccurate estimation of *D*_56_ for our special case, when negative branch lengths are not allowed, is illustrated in Fig. 2b. I highlight two points. First, when *D_ij_* are accurate, then the FM criterion is better than the ME criterion (e.g. when *D*_56_ is within the range of 0.19–0.21 in Fig. 2). Second, when the error associated with *D_ij_* is sufficiently large, then at least the FM2 criterion is worse than the ME criterion (e.g. when *D*_56_ is < 0.17 or > 0.25 in Fig. 2). While previous simulations suggest that disallowing negative branches may significantly improve the performance of distance-based methods (Kuhner and Felsenstein, 1994), our results show that the improvement may only be seen in cases where *D_ij_* values are estimated with great accuracy.

The various biases under different conditions are summarized in Table 2 to facilitate cross-reference.

## Simulation and Discussion

**Figure 3:**
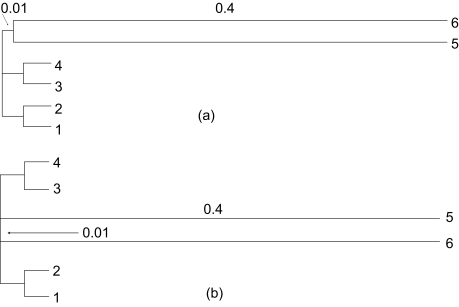
Two contrasting model trees used to simulate sequence evolution. All branch lengths are 0.02 except for those specifically labeled.

Given the topological bias identified in the previous section, I used simulation to further explore the extent of the bias in practical scenarios. I used the EVOLVER program in the PAML package (http://abacus.gene.ucl.ac.uk/software/paml.html) to simulation sequence evolution with Tree A and Tree B in Fig. 3 which correspond to Topology A and Topology B (Fig. 1), respectively. Phylogenetic analysis was carried out by using DAMBE (Xia, 2001; Xia and Xie, 2001).

The substitution model used in the simulation is *K*80 (Kimura, 1980), with κ = 5 and equal nucleotide frequencies, and with no rate heterogeneity over sites. The sequence length was set to 1500 bases and the simulation of sequence evolution was repeated 500 times for each tree.

I used the JC69 and *F*84 distances to compute genetic distances. Because the sequences are generated with the *K*80 model, *D_JC_* will tend to underestimate *D_K_*_80_ when Q ≠ 2P (where P and Q are the proportion of sites with transitional and transversion substitutions, respectively) due to stochastic noise, and the resulting tree is expected to exhibit the bias associated with underestimation of *D_ij_*. In contrast, *D_F_*_84_ tends to overestimate *D_K_*_80_ whenever nucleotide frequencies are different from 0.25 and the resulting tree is expected to exhibit the bias associated with overestimation of *D_ij_*.

### Simulation with Tree A

#### Underestimation of *D*_56_

**Figure 4:**
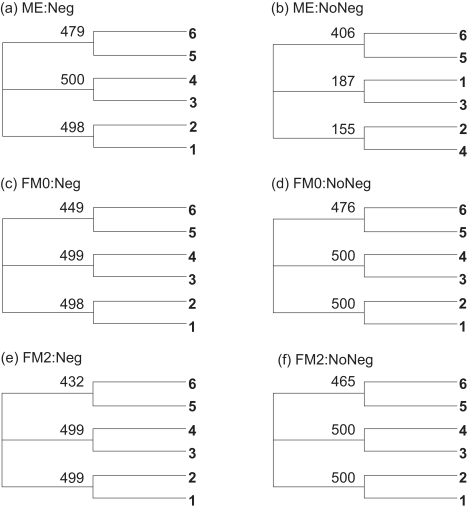
Consensus trees from simulated sequences using the *K*80 model and Tree A in Fig. 3 but with *D_ij_* estimated by the JC69 model. The label for each tree is in the form of “Criterion:Negative branch lengths allowed or not.”

For sequences generated with the two-parameter *K*80 model and Tree A in Fig. 3, but *D_ij_* is estimated with *D_JC_*, *D_ij_* values are biased towards underestimation. In particular, *D*_56_ tends to be most severely underestimated. According to Table 2, the underestimated *D*_56_ should favor Topology A, regardless of which of the ME and FM criteria is used and whether negative branch lengths are allowed. Note that the bias here actually favors the recovery of the true tree. The six consensus trees from 500 sets of sequences (Fig. 4) are consistent with the prediction.

There is substantial difference in bootstrap values between the consensus tree in Fig. 4a (with negative branch length allowed), and that in Fig. 4b (with no negative branch length allowed), based on the ME criterion. This is expected from Table 2. With an underestimated *D*_56_, the ME criterion is more biased towards Topology A when negative branch lengths are allowed than when negative branch lengths are not allowed (Table 2). So we expect the bootstrap support for Topology A to be stronger when negative branch lengths are allowed (Fig. 4a) than when negative branch lengths are not allowed (Fig. 4b).

#### Overestimation of *D*_56_

**Figure 5:**
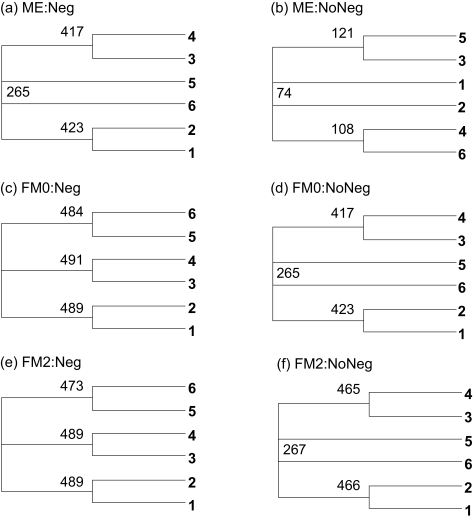
Consensus trees from simulated sequences using the *K*80 model and Tree A in Fig. 3 but with *D_ij_* estimated by the *F*84 model. The tree label format is the same as Fig. 4.

For the same 500 sets of sequences generated with the *K*80 model and Tree A, but *D_ij_* estimated with the *F*84 model, *D_ij_* values (especially *D*_56_) are biased towards overestimation. Table 2 suggests two predictions. First, the overestimated *D*_56_ should favor Topology B with the ME criterion, regardless of whether negative branches are allowed or not. Second, with the FM criterion, Topology A is favored if negative branches are allowed, but Topology B is favored if negative branch lengths are not allowed. These two predictions are supported (Fig. 5). First, both consensus trees based on the ME criterion (Fig. 5a and b, one with negative branches allowed and the other not) assume Topology B. Second, for the FM criterion, the consensus tree allowing negative branches (Fig. 5c and e) exhibit Topology A, whereas the two consensus trees (Fig. 5d and f) not allowing negative branches have Topology B. For the consensus trees in Fig. 5d and Fig. 5f, the bootstrap values between OTUs 5 and 6 (265 and 267, respectively, in Fig. 5d and Fig. 5f) are mainly due to the fact that, among the 500 trees, OTU 5 is positioned closer to OTUs 1 and 2 about half of the time and to OTUs 3 and 4 another half of the time. The tree shape (Felsenstein, 2004, p. 33) is strongly supported.

### Simulation with Tree B

#### Underestimated *D*_56_

**Figure 6:**
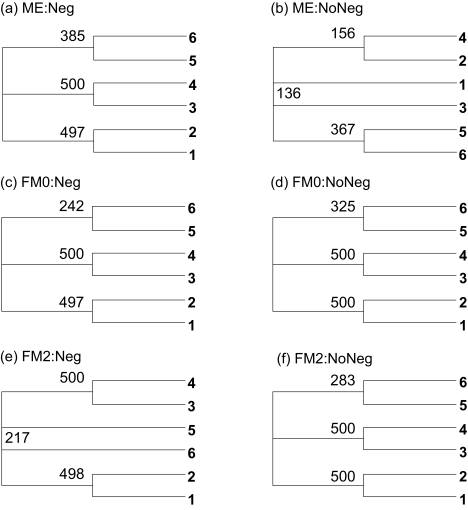
Consensus trees from simulated sequences using the *K*80 model and Tree B in Fig. 3 but with *D_ij_* estimated by the JC69 model. The tree label format is the same as Fig. 4.

For sequences generated with the two-parameter *K*80 model, but *D_ij_* is estimated with *D_JC_*, *D_ij_* values are biased towards underestimation. In particular, *D*_56_ tends to be most severely underestimated. According to Table 2, the underestimated *D*_56_ should favor Topology A even the model tree has Topology B, regardless of which of the ME and FM criteria is used and whether negative branch lengths are allowed. The consensus trees from 500 sets of sequences (Fig. 6) substantiate this prediction. It is remarkable that, while the model tree has Topology B, most reconstructed trees recovered Topology A (Fig. 6 a, c, d, f). This is a powerful demonstration of the topological bias.

The bias associated with the ME criterion in favor of Topology A should be alleviated by not allowing negative branches (Table 2). This explains why the consensus tree allowing negative branches (Fig. 6a) assumes Topology A whereas the consensus tree not allowing negative branches (Fig. 6b) assumes Topology B. We also note that not allowing negative branches makes the FM2 criterion even more biased in favor of Topology A (Table 2). This explains why the consensus tree in Fig. 6f assumes Topology A whereas the consensus tree in Fig. 6e assumes Topology B.

#### Overestimated *D*_56_

**Figure 7:**
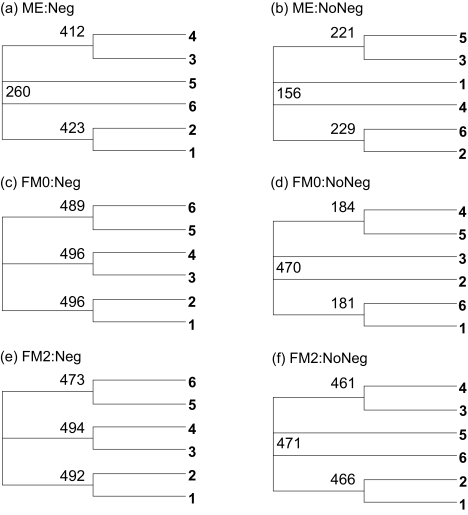
Consensus trees from simulated sequences using the *K*80 model and Tree B in Fig. 3 but with *D_ij_* estimated by the *F*84 model. The tree label format is the same as Fig. 4.

For the same 500 sets of sequences generated with the *K*80 model and Tree B in Fig. 3, but *D_ij_* estimated with the *F*84 model, *D_ij_* values (especially *D*_56_) are biased towards overestimation. According to Table 2, the overestimated *D*_56_ should favor Topology B with the ME criterion. This is supported because both consensus trees (Fig. 7a and b) exhibit Topology B.

For the FM criterion, the topological bias associated with the overestimation of *D*_56_ will be in opposite direction depending on whether negative branch lengths are allowed or not. According to Table 2, the FM criterion will favor Topology A when negative branch lengths are allowed, but Topology B when negative branch lengths are not allowed. This prediction is clearly substantiated by the consensus trees obtained with the FM criterion, especially with the FM2 criterion (Fig. 7e–f), where the consensus tree assumes Topology A when negative branch lengths are allowed but Topology B when negative branch lengths are not allowed.

I should finally discuss four issues closely related to our study. First, our result highlights the inadequacy of many studies evaluating relative performance of phylogenetic algorithms involving only four OTUs. The topological bias in the distance-based methods shown in our paper cannot be revealed unless one has at least six OTUs because, for unrooted trees with fewer OTUs, there is only one unrooted bifurcating tree shape.

Second, when a large number of OTUs are included in a phylogenetic study, it is highly likely that different subtrees may be governed by different substitution models and the genetic distance based on any one particular distance may overestimate some distances and underestimate others. This may cause problems in building very large trees with different subtrees suffering from different topological biases.

Third, the current paper is limited in at least two major ways. First, much of the analysis is based on the consequence of overestimation or underestimation of *D*_56_. Second, it does not provide a large-scale and realistic simulation to explore the consequence of the bias in practice. We are currently addressing these two problems.

## Figures and Tables

**Table 1:** The effect of inaccurate estimation of *D*_56_ (= *x*_8_ + *x*_9_) when the true tree is Topology C in Fig. 1 with all branch lengths equal to 0.1, except for *x*_8_ and *x*_9_. The *x*_7_ values are from the least-squares evaluation with Topology A. *T_b.ME,_* *T_b.FM0_* and *T_bFM2_* are explained in the text.

x_8_,x_9_	D_56_	T_b.ME_	T_b.FM0_	T_b.FM2_	x_7_
0.0800	0.16	−0.01111	−0.00071	−0.01635	0.0200
0.0900	0.18	−0.00556	−0.00018	−0.00344	0.0100
0.1000	0.20	0.00000	0.00000	0.00000	0.0000
0.1100	0.22	0.00556	−0.00018	−0.00263	−0.0100
0.1200	0.24	0.01111	−0.00071	−0.00949	−0.0200
0.1300	0.26	0.01667	−0.00160	−0.01953	−0.0300

**Table 2:** Summary of topological biases showing which of the two topologies (A and B) is favored under different conditions. Strong bias is indicated by bold font.

	Neg. branch(1)	ME	FM0	FM2
Underestimated *D*_56_	Allowed	**A**	A	A
	Not allowed	A	A	**A**
Overestimated *D*_56_	Allowed	**B**	A	A
	Not allowed	B	B	B

(1) Negative branches.
